# Supplementary material for: Sustained-input switches for transcription factors and microRNAs are central building blocks of eukaryotic gene circuits
Source: Genome Biol. 2013 Aug 23;14(8):R85. doi: 10.1186/gb-2013-14-8-r85 (PMC4054853; doi:10.1186/gb-2013-14-8-r85)
Supplement: Additional file 5 — HTML Browsable Motif Output. Zipped folder containing all WaRSwap and FANMOD motif output, viewable in a web browser. [file gb-2013-14-8-r85-S5.ZIP › HTML_browsable_motif_output/FANMOD_ath_tair9/sigs_fanmodm-2000.pvals.heatmaps.html/motif_id_6_000111002_tftype_ath_upstream_-1000_0.html]

```
BG_MODEL = FANMOD
MOTIF_ID = 6_000111002
TF_TYPE = ath
UPSTREAM = -1000_0


PVals
FN_0.2	FN_0.4	FN_0.6	FN_0.8
dg_60.genes	0.227	0.225	0.11	0.103
dg_70.genes	0.422	0.48	0.168	0.096
dg_80.genes	0	0	0	0

ZScores
FN_0.2	FN_0.4	FN_0.6	FN_0.8
dg_60.genes	1.084	1.197	1.416	1.375
dg_70.genes	0.68	0.76	1.068	1.206
dg_80.genes	2.501	2.051	1.3	1.089

StDevs
FN_0.2	FN_0.4	FN_0.6	FN_0.8
dg_60.genes	384.911	231.502	76.291	25.897
dg_70.genes	232.623	133.767	46.604	14.304
dg_80.genes	4.981	4.99	5.658	2.523
```
